# Does total volume of physical activity matter more than pattern for onset of CVD? A prospective cohort study of older British men

**DOI:** 10.1016/j.ijcard.2018.12.024

**Published:** 2019-03-01

**Authors:** Barbara J. Jefferis, Tessa J. Parsons, Claudio Sartini, Sarah Ash, Lucy T. Lennon, Olia Papacosta, Richard W. Morris, S. Goya Wannamethee, I-Min Lee, Peter H. Whincup

**Affiliations:** a Department of Primary Care & Population Health, University College London, Rowland Hill Street, London, NW3 2PF, UK; b Population Health Sciences, Bristol Medical School, University of Bristol, Canynge Hall, 39 Whatley Road, Bristol BS8 2PS, UK; c Harvard Medical School, Brigham and Women's Hospital, 900 Commonwealth Avenue East, Boston, MA02215, USA; d Population Health Research Institute, St George's University of London, Cranmer Terrace, London, SW17 0RE, UK

**Keywords:** Physical activity, Sedentary behaviour, Accelerometer, CHD, Stroke, Heart failure

## Abstract

**Aims:**

With increasing age, physical inactivity and sedentary behaviour levels increase, as does cardiovascular disease (CVD) incidence. We investigate how device-measured sedentary behaviour and physical activity (PA) are related to CVD onset in men aged 70+; whether the total volume of activity is more important than pattern.

**Methods and results:**

Prospective population-based cohort study of men recruited from 24 UK General Practices in 1978–80. In 2010–12, 3137 survivors were invited to complete questionnaires and wear an Actigraph GT3x accelerometer for 7 days. PA intensity was categorised as sedentary, light and moderate to vigorous (MVPA). Men were followed up for Myocardial Infarction, stroke and heart failure (ICD9 410–414, 430–438 and 428) morbidity and mortality from 2010 to 12 to June 2016. Hazard Ratios (HRs) for incident Cardiovascular Disease (CVD) were estimated. 1528/3137 (49%) men had sufficient accelerometer data. 254 men with pre-existing CVD were excluded. Participants' mean age was 78.4 (range 71–92) years. After median 4.9 years follow-up, 122 first CVD events occurred in 1181 men (22.7/1000 person-years) with complete data. For each additional 30 min in sedentary behaviour, light PA,10 min in MVPA, or 1000 steps/day, HRs for CVD were 1.09(95%CI 1.00, 1.19), 0.94(0.85, 1.04), 0.88(0.81, 0.96) and 0.86(0.78 to 0.95) respectively, adjusted for measurement-related factors, socio-demographics, health behaviours and disability. HRs for accumulating 150 min/week MVPA in bouts ≥1 min and bouts ≥10 min were 0.47(0.32 to 0.69), and 0.49(0.25, 0.98).

**Conclusions:**

In older men, high volume of steps or MVPA rather than MVPA bouts was associated with reduced CVD risk.

## Highlights

• Prospective studies report that self-reported physical activity protects against cardiovascular disease (CVD).
• Yet, self-reported behaviours cannot easily be used to assess the value of the 10 min activity bouts specified in guidelines.
• This is the first study of risk of fatal and non-fatal CVD based on device measured physical activity and sedentary behaviour.
• Among 1181 men aged over 70 years, more time in moderate to vigorous physical activity and more steps reduced 5-year CVD risk.
• The pattern of accumulating moderate to vigorous physical activity in 10 min bouts was not important for CVD risk.

## 1 Introduction

Physical activity (PA) is a key preventive measure against cardiovascular disease (CVD): with 25–30% reductions in risk of CVD events in middle aged adults doing moderate compared to low PA [1]. However most evidence underpinning PA guidelines is derived from epidemiologic studies linking self-reported activity data to onset of CVD events and mortality [2].

Current guidelines [2,3] suggest accumulating moderate to vigorous physical activity (MVPA) in 10 min bouts, based on trial data for cardiometabolic risk factors only, not clinical end points [2]. However it is not clear whether the total volume of activity is critical for prevention of CVD events, or if 10 min bouts are necessary. Studies using self-reported PA are not well placed to answer this question. However the availability of body-worn sensors in large scale epidemiologic studies enables measurement of the pattern of accumulation of activities and the number of minutes per day spent in different intensities of activity. Importantly, this includes light activity (the most common intensity), which can be hard to recall.

Many studies report that higher levels of self-reported sedentary time are associated with CVD onset [4], although self-report sedentary behaviours are prone to measurement error or recall bias [5,6]. Experimental studies suggest benefits of breaking up sedentary time to improve levels of metabolic and hemostatic markers [7,8]. Hence activity guidelines now suggest avoiding “long” sedentary periods [3]. However it is unclear how much sedentary behaviour and in what patterns, is associated with onset of CVD events.

To date, no published studies with device-measured PA and sedentary behaviour data have investigated how PA is related to incident fatal and non-fatal CVD events. Using a cohort of older British men, we investigated (i) how device-measured PA and sedentary behaviour were related to incident CVD; (ii) the strength and shape of dose-response associations, to better understand whether the reductions in CVD risk with increasing PA levels are linear, or if there was a threshold level at which the benefits per unit of activity decrease; (iii) whether the association of sedentary behaviour with CVD risk depended on PA level; and (iv) associations between patterns of accumulation of activity (including bout length, and sedentary breaks) and CVD. Older adults are an understudied population yet they are important because they are growing rapidly and the burden of CVD increases with age, whilst PA levels decline rapidly.

## 2 Methods

### 2.1 Study population

The British Regional Heart Study (BRHS) is a prospective cohort study of 7735 men recruited from a single General Practice in each of 24 British towns in 1978–80 (age 40–59 years). In 2010–2012, survivors (*n* = 3137) were invited to a physical re-examination including objective PA assessment [9]. The National Research Ethics Service Committee London provided ethical approval, men provided informed written consent in accordance with the Declaration of Helsinki.

### 2.2 Measurements at 2010–2012 examination

#### 2.2.1 Objective physical activity assessment

Men were invited to wear a GT3x accelerometer (Actigraph, Pensacola, FL. USA) over the right hip for 7 days, during waking hours, except for bathing and swimming (2% reported swimming). Accelerometer data was processed using standard methods [10] and non-wear time was excluded using the R package “Physical Activity” [10,11]. Participants with ≥600 min wear time, on ≥3 days were included, a conventional requirement for estimating usual PA level [10,[12], [13], [14]]. Each minute of wear time was categorised using intensity threshold values of counts per minute (CPM) from the vertical axis developed for older adults: <100 for sedentary behaviour (<1.5 MET), 100–1040 for light activity (LIPA) (1.5–3 MET) and >1040 for MVPA, (≥3 MET) [15].

#### 2.2.2 Nurse measured CVD risk factors

Nurses took fasting blood samples; low density lipoprotein cholesterol (LDLC) was adjusted for duration of fasting and time of day. Systolic blood pressure was measured twice (Omron recorder, mm Hg) and the average of 2 readings used, adjusted for cuff size. The Chronic Kidney Disease Epidemiology Collaboration Creatinine-Cystatin (CKD-EPI cr-cys) equation was used to calculate estimated Glomerular Filtration Rate (eGFR) [16]. Chronic Kidney Disease was defined as eGFR<45 ml/min per 1.73 m^2^ [16]. BMI, (kg/m^2^) was calculated from measured height (Harpenden stadiometer) and weight (Tanita body composition analyser (BC-418-MA)) in light indoor clothing.

#### 2.2.3 Questionnaire data

Men self-reported current cigarette smoking, alcohol consumption, usual night-time sleep duration, living alone or with others, and doctor diagnosis of type 2 diabetes, atrial fibrillation, pre-existing CVD (heart attack, heart failure or stroke (with symptoms lasting >24 h)), and use of medications including antihypertensives (British National Formulary (BNF) codes 2.2.1, 2.2.8, 2.4, 2.5.5 and 2.6.2) or statins (BNF 2.12). Mobility disability was present if participants were unable to do any of (i) walk 200 yards without stopping and without discomfort (ii) climb a flight of 12 stairs without holding on and taking a rest or (iii) bend down and pick up a shoe from the floor. Social class was based on longest held occupation at study entry (1978–80) (categorised as manual and non-manual). Region of residence (1978–80) was grouped into Scotland, North, Midlands and South of England.

#### 2.2.4 CVD events and mortality

All men were followed up for all-cause mortality and first fatal or non-fatal Myocardial Infarction, stroke or heart failure event (herein referred to as CVD), occurring between the 2010–2012 survey and 1st June 2016. All deaths were ascertained through the National Health Services Central Registers. Deaths were identified from International Classification of Disease (ICD) 9 codes: MI 410–414 (ICD 10 codes I20–I25), Stroke 430–438, (ICD10 I60–69) and Heart Failure 428 (ICD10 I50). Nonfatal events were recorded from yearly reviews of primary care notes (which include correspondence and diagnoses from secondary care). MI was reported as heart attack or coronary thrombosis, diagnosed in accordance with WHO criteria [17] and stroke events were those that produced a neurological deficit present for >24 h. Physician diagnosis of Heart failure was verified using available clinical information (including symptoms, signs, investigations, and treatment response) consistent with current diagnostic practice [18]; cases with a strong likelihood of alternative diagnoses were excluded. All cases were adjudicated by study director (PHW).

### 2.3 Statistical methods

Means, medians or proportions of covariates selected a priori were compared among quartiles of MVPA. Hazard Ratios (HRs) for CVD according to (i) total steps per day and total daily minutes in (ii) MVPA (iii) LIPA and (iv) sedentary behaviour were estimated using Cox Proportional Hazards models. Each activity measure was analysed in quartiles and then as a continuous variable. To facilitate interpretation, HRs were estimated for each increase of 1000 steps/day, 30 min of sedentary behaviour or LIPA and 10 min of MVPA. Survival times were censored at death (any cause) or 1 June 2016. Models were cumulatively adjusted as follows: Model 1 included age, region of residence, average accelerometer wear time (minutes/day), season of wear (May–September vs October–April). Model 2; additionally: social class, living alone, sleep duration, smoking status, alcohol consumption and BMI. Model 3; additionally: presence of mobility disability. Model 4; additionally: another intensity of PA to investigate whether (i) MVPA and sedentary behaviour and (ii) MVPA and LIPA were associated with CVD independently of each other. We compared model fit between linear and quadratic models (using a likelihood ratio test), to test the shape of association between activity and CVD based on a priori expectations about the shape of association [19]. The shapes of associations were checked using penalised splines (a non-parametric estimation method which makes few assumptions about the underlying shape of the association). Predicted values from spline models were plotted. To assess model fit, the Akaike information criterion (AIC) was compared between linear and spline models.

We estimated the HR for CVD for men who accumulated ≥150 min MVPA/week (vs <150 min) in bouts lasting (i) ≥1 and (ii) ≥10 consecutive minutes (current recommendation [3]. Next we estimated the HR for CVD per minute of MVPA in bouts lasting (i) 1–9 min and (ii) ≥10 min, and tested the difference between the two coefficients. Models were repeated for light PA. For sedentary behaviour we compared bouts lasting 1–15 min, 16–30, 31–60 and over 60 min. We estimated the HR for CVD for the number of sedentary breaks/hour, (defined as the interruption of a sedentary bout lasting >1 min by ≥1 min of LIPA or MVPA). Number of sedentary breaks per hour was analysed in quartiles and models were adjusted for total sedentary time.

Sensitivity analyses (see Web Appendix), investigated (i) the non-normal distribution of MVPA, (ii) the percentage of the day spent in each activity (iii) excluding the first year of follow-up and (iv) excluding the first year of follow-up and men with disability (v) confounding by socio-economic status (vi) confounding by other co-morbidities (vii) additional adjustments for lipids, blood pressure and lipid and blood pressure lowering medications. The impact of missing co-variate data was investigated by using multiple imputation in v, vi and vii. Interactions were investigated between sedentary behaviour and PA level, and secondly between each of MVPA, LIPA and sedentary behaviour and age and adiposity.

## 3 Results

Of 3137 surviving men, 1566 (50%) agreed to participate and returned an accelerometer with data. A comparison of included and excluded participants characteristics reported 10 years previously is in Supplementary Table 1: men with Actigraph data tended to be younger, from non-manual social class, more active, weighed less but were of similar height to men without Actigraph data. Of the 1566 men with Actigraph data, 1528 (49%) had ≥600 min/day wear time on ≥3 days. 254 men with pre-existing heart attack, heart failure or stroke were excluded. Of the remaining 1274, participants' mean age was 78.4 (range 71–92) years. Mean accelerometer wear time was 855 min/day, of which 616 min were in sedentary behaviour and 199 in LIPA and 40 in MVPA. The distribution of MVPA minutes was right-skewed. The distribution of bouts spent in each intensity of activity is in Supplementary Table 2.

There were dose-response associations across quartiles of total MVPA; men who were more active compared to less active were younger, less likely to smoke cigarettes, and had greater alcohol consumption, lower mean BMI, prevalence of mobility disability, CKD, diabetes, atrial fibrillation, and use of lipid-and BP lowering medications and spent less time in sedentary behaviour (Supplementary Table 3). Opposite dose-response associations were observed for sedentary behaviour quartiles (data not presented).

### 3.1 PA, sedentary behaviour and CVD events

During a median follow-up of 4.9 years (range 0.1–6.1), 122 CHD, stroke or heart failure events occurred (80 non-fatal), (22.6/1000 person-years) among 1181 men with complete case data. The risk of CVD events was lower in the higher quartiles of steps (Table 1) and MVPA (Table 4) and higher in the lowest quartile of sedentary time (Table 2). There were no clear dose-response associations across quartiles of light PA (Table 3). We did not find statistical evidence for better fit for quadratic than linear models for any of the PA measures in relation to CVD events, nor did splines support non-linear associations (see Web Appendix 1, Figs. 1–4 and Table 4). For each additional 1000 steps/day the HR for CVD events was 0.83 (95% CI 0.75, 0.91) (Model 1, Table 1). For each additional 30 min in sedentary behaviour and LIPA or 10 min in MVPA, HRs (Model 1) were 1.14 (1.06, 1.24) (Table 2), 0.89 (0.80, 0.98) (Table 3) and 0.85 (0.79, 0.93) (Table 4) respectively. Adjustments for socio-demographic factors, health behaviours and sleep time (Model 2) and mobility disability (Model 3) did not appreciably affect estimates and CIs for steps and MVPA but completely attenuated the association for light PA. In Model 4 adjustment for MVPA and sedentary time (Model 4), the coefficient for sedentary time was fully attenuated (Table 2) whereas the MVPA coefficient was little changed (Table 4).

**Table 1:** Association Between Steps per Day With CVD events, (n=1181 Men).

	Quartile 1 (121–2943)	Quartile 2 (2944–4540)		Quartile 3 (4541–6406)		Quartile 4 (6407–17,781)		All men, per 1000 steps^d^	
N Participants (n CVD events)	296 (53)	295 (35)		295(20)		295 (14)		1181 (122)	
Person years	1214	1342		1398		1425		5378	
CVD events/ 1000 person years	43.6	26.1		14.3		9.8		22.6	

		HR	95%CI	HR	95%CI	HR	95%CI	HR	95%CI
Model 1^a^	Reference	0.65	(0.41,1.01)	**0.36**	**(0.21,0.63)**	**0.27**	**(0.14,0.52)**	**0.83**	**(0.75,0.91)**
Model 2^b^	Reference	0.67	(0.43,1.06)	**0.39**	**(0.23,0.68)**	**0.3**	**(0.15,0.59)**	**0.84**	**(0.76,0.93)**
Model 3^c^	Reference	0.75	(0.47,1.20)	**0.44**	**(0.25,0.77)**	**0.34**	**(0.17,0.67)**	**0.86**	**(0.78,0.95)**

Bold text indicates p<0.05.

a model 1 = age + region of residence + season of wear + accelerometer wear time.

b model 2 = model 1 + social class + alcohol use + smoking + sleep time + living alone + BMI.

c model 3 = model 2 + mobility disability.

d HR for mortality per 1000 steps per day (continuous variable).

**Table 2:** Association Between Minutes per Day in Sedentary Behaviour With CVD events, (n=1181 Men).

	Quartile 1 (295–560)	Quartile 2 (561–615)		Quartile 3 (616–671)		Quartile 4 (672–1054)		All men, per 30 min^e^	
N Participants (n CVD events)	296 (24)	295 (23)		296 (33)		294 (42)		1181 (122)	
Person years	1417	1381		1341		1249		5378	
CVD events/ 1000 person years	17.1	16.7		24.6		33.6		22.6	

		HR	95%CI	HR	95%CI	HR	95%CI	HR	95%CI
Model 1^a^	Reference	0.98	(0.55,1.74)	1.42	(0.82,2.47)	**2.18**	**(1.24,3.83)**	**1.14**	**(1.06,1.24)**
Model 2^b^	Reference	0.92	(0.52,1.65)	1.32	(0.76,2.31)	**1.87**	**(1.03,3.38)**	**1.12**	**(1.03,1.21)**
Model 3^c^	Reference	0.90	(0.50,1.60)	1.20	(0.68,2.10)	1.63	(0.90,2.97)	**1.09**	**(1.00,1.19)**
Model 4^d^	Reference	0.70	(0.38,1.28)	0.81	(0.43,1.52)	0.94	(0.45,1.94)	1.01	(0.90,1.13)

Bold text indicates p<0.05.

a model 1 = age + region of residence + season of wear + accelerometer wear time.

b model 2 = model 1 + social class + alcohol use + smoking + sleep time + living alone + BMI.

c model 3 = model 2 + mobility disability.

d model 4 = model 3 + MVPA.

e HR for mortality per 30 minutes of sedentary behaviour per day (continuous variable).

**Table 3:** Association Between Minutes per Day in Light Physical Activity With CVD events, (n=1181 Men).

	Quartile 1 (5–157)	Quartile 2 (158–197)		Quartile 3 (198–238)		Quartile 4 (239–472)		All men, per 30 minutes^e^	
N Participants (n CVD events)	296 (45)	295 (34)		296 (19)		294 (24)		1181 (122)	
Person years	1233	1318		1428		1400		5378	
CVD events/ 1000 person years	36.5	25.8		13.3		17.1		22.6	

		HR	95%CI	HR	95%CI	HR	95%CI	HR	95%CI
Model 1^a^	Reference	0.75	(0.47,1.19)	**0.41**	**(0.23,0.72)**	0.58	(0.33,1.02)	**0.89**	**(0.80,0.98)**
Model 2^b^	Reference	0.78	(0.49,1.24)	**0.45**	**(0.26,0.80)**	0.67	(0.38,1.20)	0.92	(0.83,1.02)
Model 3^c^	Reference	0.86	(0.53,1.38)	**0.49**	**(0.28,0.87)**	0.74	(0.41,1.34)	0.94	(0.85,1.04)
Model 4^d^	Reference	0.93	(0.57,1.50)	0.59	(0.33,1.06)	0.97	(0.53,1.80)	0.99	(0.89,1.11)

Bold text indicates p<0.05.

a model 1 = age + region of residence + season of wear + accelerometer wear time.

b model 2 = model 1 + social class + alcohol use + smoking + sleep time + living alone + BMI.

c model 3 = model 2 + mobility disability.

d model 4 = model 3 + MVPA.

e HR for mortality per 30 minutes of LIPA per day (continuous variable).

**Table 4:** Association Between Moderate to Vigorous Physical Activity (MVPA) With CVD events, (n=1181 Men).

	Quartile 1 (0.4–15)	Quartile 2 (16–32)		Quartile 3 (33–56)		Quartile 4 (57–187)		All men, per 10 minutes^e^	
N Participants (n CVD events)	297 (57)	296 (33)		294 (15)		294 (17)		1181 (122)	
Person years	1222	1348		1395		1414		5378	
CVD events/ 1000 person years	46.7	24.5		6.5		7.5		22.6	

		HR	95%CI	HR	95%CI	HR	95%CI	HR	95%CI
Model 1^a^	Reference	**0.56**	**(0.36,0.87)**	**0.26**	**(0.14,0.46)**	**0.31**	**(0.17,0.55)**	**0.85**	**(0.79,0.93)**
Model 2^b^	Reference	**0.58**	**(0.37,0.91)**	**0.27**	**(0.15,0.49)**	**0.34**	**(0.18,0.62)**	**0.87**	**(0.80,0.95)**
Model 3^c^	Reference	0.63	(0.39,1.01)	**0.29**	**(0.16,0.55)**	**0.37**	**(0.20,0.68)**	**0.88**	**(0.81,0.96)**
Model 4^d^	Reference	0.62	(0.38,1.00)	**0.28**	**(0.14,0.55)**	**0.34**	**(0.16,0.74)**	**0.89**	**(0.80,0.99)**

Bold text indicates p<0.05.

a model 1 = age + region of residence + season of wear + accelerometer wear time.

b model 2 = model 1 + social class + alcohol use + smoking + sleep time + living alone + BMI.

c model 3 = model 2 + mobility disability.

d model 4 = model 3 + sedentary behaviour.

e HR for mortality per 10 minutes of MVPA per day (continuous variable).

### 3.2 Bouts of activity and CVD events

The HR in Model 1 for accumulating ≥150 min MVPA/week accumulated in sporadic minutes (achieved by 66% of men) compared to <150 min, was 0.47 (0.32 to 0.69), and 0.49(0.25, 0.98) for accumulating 150 min MVPA/week all in bouts lasting ≥10 min (achieved by 16% of men). Estimates for 1 min bouts were not meaningfully changed in models 2 or 3, but were attenuated for 10 min bouts in models 2 and 3 (Supplementary Table 5).

The HRs for CVD for each minute of MVPA spent in bouts lasting 1–9 min did not differ from the HR for each minute of MVPA spent in bouts lasting ≥10 min (*p* = 0.75), nor for LIPA (*p* = 0.44) (Supplementary Table 6). The number of minutes spent in sedentary bouts lasting 1–15 min, 16–30, 31–60 and >61 min were all similarly associated with CVD (Supplementary Table 6). The HR for CVD did not differ significantly between quartiles of sedentary breaks per hour (Supplementary Table 7). Web Appendix 1 presents sensitivity analyses (relating to the distribution of MVPA, further exclusions and confounding by SES, additional co-morbidities and adjustments for variables on the pathway between PA and CVD, as well as missing data imputation); these did not change our main conclusions, except that in model 4 the association between MVPA and CVD was attenuated by adjustment for sedentary time. Interactions were not observed between physical activity level, age, adiposity and disability (Web Appendix 1).

## 4 Discussion

Consistent prospective associations between higher total daily step count and minutes spent in MVPA and lower risk of CVD events were observed in community-dwelling older men. Associations changed little after adjustment for other health behaviours, BMI, and presence of mobility disability. After full adjustment the associations between quartiles of MVPA and CVD remained consistently strong, with for example a 72% reduction in risk of CVD events for men doing 33–56 min/day MVPA compared to ≤15 min/day. Investigating the shape of associations between total minutes/day spent in MVPA and steps/day with CVD indicated that the lower CVD risks were gained across the spectrum of step counts and time in MVPA, not confined to a threshold duration. Further, the total volume rather than pattern of accrual of physical activity was the most important influence on CVD risk.

Our data extends evidence about device measured physical activity and CVD to include non-fatal as well as fatal CHD, stroke and heart failure events, other studies only investigated CVD mortality or defined CVD more broadly [[20], [21], [22], [23]]. We provide evidence on an older population (72–91 years at baseline), important given the sparsity of data on men over 80 [21,22], and on a non-US population (all other reports of CVD [[20], [21], [22], [23]] and all-cause mortality are restricted to USA data [[22], [23], [24], [25], [26], [27], [28], [29], [30]], and nearly all use one data source). Prior studies did not report tests of non-linearity in associations between device-measured activity and CVD mortality, nor specific bouts of LIPA, and breaks in sedentary time and only one investigated bouts of SB and MVPA [23].

### 4.1 PA intensity and duration

Comparing results to other studies using objective PA data is difficult because definitions of activity intensity and analysis methods vary. We found each increase of 1000 steps/day was associated with a 14% reduction in CVD risk, but other studies have not reported comparable data. Further, each 10 min/day increase in MVPA was associated with an 11% reduction in CVD risk (approximately 85% reduction per hour of MVPA), which was not explained by adjustment for behavioural and social confounders and mobility disability. Whilst Evenson et al. using NHANES data report that CVD mortality was lower in the highest three quartiles of moderate PA and of MVPA than the lowest quartile, there did not appear to be a dose-response association, but MVPA was defined using a higher cut-point (>2020 CPM) in their younger population [23].

We did not find associations between LIPA and CVD risk, nor did Evenson's NHANES study [23], although in a different analysis of NHANES data Schmid et al. found that replacing sedentary time with LIPA was associated with lower CVD mortality [20]. Another study of older men [22] found less consistent associations between LIPA and lower CVD mortality. Whereas, a study of older women reported that LIPA was associated with lower CVD mortality, even after adjustment for health and social confounders [21]. In the latter two studies, the cut-points defining LIPA are not directly comparable to ours.

We found that adjustment for MVPA fully explained the association between higher sedentary time and raised CVD risk. We did not find significant associations of sedentary breaks with CVD events. Other studies [22,23] did not find consistent associations between minutes/day spent in SB and CVD mortality, but Evenson's study did find that more time spent in longer SB bouts was associated with increased CVD mortality [23], albeit in a slightly younger population. Although self-reported sedentary time (often television viewing time) is associated with CVD risk [4], reasons for contrasting results may include the difference in objective vs self-report measures [31], confounders related to TV viewing, as well as the older age group studied here (the strength of other classic risk factors for CVD declines at older ages [32]).

### 4.2 Linearity of associations

Our results suggest that more daily steps and minutes of MVPA are beneficial, rather than benefits only accruing above a particular threshold. Whilst setting activity targets may be useful as a behavioural change tool, at least for CVD, we don't have a clear evidence base for a target. Nevertheless, accumulating more than around 30 min total MVPA (not necessarily in 10 min bouts), or 4500 steps per day were associated with lower CVD risks than the lowest quartiles of those measures. Although other studies report results for tertiles or quartiles rather than specifically testing for non-linear continuous associations, NHANES results suggested linear dose-response only for the average CPM rather than for MVPA or LIPA [23].

### 4.3 Pattern of activity: bouts and breaks

Many PA guidelines advise accumulating MVPA in bouts lasting ≥10 min and avoiding long sedentary bouts [2,3]. We did not find clear evidence that sedentary breaks were associated with mortality risk after accounting for total sedentary time. Overall our results suggested that the total time spent in MVPA rather than pattern of bouts was important. The only other study to investigate bouts in relation to CVD risk reported some suggestive evidence that more time spent in MVPA bouts ≥10 min was associated with lower CVD mortality, but the association was non-linear and their bout definition less strict (allowing ≤5 min of lower intensity activity within bouts [23]). Hence for CVD prevention among older men, accumulating ≥10 min bouts of activity may not be essential; it may be better for older adults to focus on increasing time spent in moderate (or more intense) activity.

The varied findings of studies to date indicate that that more studies are needed; inconsistencies may be due to differences in (i) population characteristics; e.g., PA levels may vary systematically (ii) age-groups (iii) devices to measure PA (iv) definitions of PA intensities (cutpoints) (v) definitions of CVD endpoints.

### 4.4 Interactions of physical activity with other variables

We did not find evidence for interactions between SB and MVPA in relation to CVD risk, as reported for all-cause mortality [33]. Nor did we find that the association between PA or sedentary behaviour and CVD onset varied by age, BMI or disability status.

### 4.5 Strengths and limitations

This study benefits from prospectively collected data on exposures, confounders, mediators and verified CVD events. Whilst we had a limited number of events (122 over 5 years among 1181 men), estimates and CIs did not differ much from models 1(fewer adjustments) to model 4, nor in sensitivity analyses where missing covariates were imputed and 137 events among 1274 men were analysed. The number of events and low number of MVPA 10 min bouts may have limited our statistical power when analysing bouts of MVPA. Whilst CHD, stroke and HF are three different types of CVD, composite end points are commonly used in the study of PA and CVD onset; furthermore a recent meta-analysis did not suggest that the relationships between self-reported physical activity and incidence of each CVD type differed [34]. We used Actigraph accelerometers to measure PA; each device was calibrated by the manufacturer prior to use in the survey. Validation data for Actigraph accelerometers is available from laboratory and field settings [15,35]. We used the conventional requirement of >3 days accelerometer wear time to estimate habitual PA levels, although 80% of participants had ≥7 days wear time. We used age-appropriate and validated cut-points to define activity intensities [15]; our lower definition of MVPA was more achievable in older adults. Our sedentary time measure may include some standing time, but the same definition performed well compared to Activpal (with postural data) in older adults [36]. The BRHS study sample was drawn from all parts of Great Britain and was nationally representative at the point of selection. 28% of all deaths were due to CVD in the BRHS over the 2010–2016 follow-up period, which is the same as the proportion in the UK male population aged 75–84 years in 2014, suggesting that the BRHS data are representative of older adults in the UK of a similar age [37]. The response rate to the accelerometer study was similar or superior to other studies of older adults [14,38], nevertheless, study participants were younger and may have been more active than the general population. However even if included participants were more active than excluded, as the sample retained a wide range of PA and sedentary behaviour levels, the association between PA and CVD should not be biased. Participants were community-dwelling older white British men, so results may not apply to women, other ethnicities or younger men, however associations between PA or sedentary behaviour and all-cause mortality did not differ by gender [24,29,30,39,40]. Results did not meaningfully change in sensitivity analyses excluding men with mobility disability, and other co-morbidities (type 2 diabetes, chronic kidney disease and atrial fibrillation), suggesting findings are not likely due to reverse causality.

## 5 Conclusions

Whilst previous work found light PA was associated with lower risk of all-cause mortality in older men [41], for CVD events, total time spent in MVPA and total steps emerged as key; accumulating activity was important, but whether it was sporadic or 10 min bouts did not matter. This suggests that among older men, it would be beneficial to promote doing more activities of an equal or greater intensity than brisk walking for protection from CVD events, rather than focusing on accumulating activity in 10 min bouts.

## Appendix A Supplementary data

Supplementary material Image 1
