# Supplementary material for: Does total volume of physical activity matter more than pattern for onset of CVD? A prospective cohort study of older British men
Source: Int J Cardiol. 2019 Mar 1;278:267–72. doi: 10.1016/j.ijcard.2018.12.024 (PMC6350006; doi:10.1016/j.ijcard.2018.12.024)
Supplement: Supplementary file 1 — Supplementary material [file mmc1.docx]

# Web Appendix

**Sensitivity analyses: Methods**

Analyses were repeated (i) using square root transformed MVPA as MVPA minutes were right skewed (ii) using percentage of the day in MVPA, LIPA and sedentary behaviour rather than minutes per day adjusted for wear time (iii) excluding the first year of follow up (during which there were 20 CVD events), to reduce the risk of reverse causality and (iv) excluding men with mobility limitations, the first year of follow up and prevalent CVD (to reduce the risk of reverse causality) (v) confounding by social class, replacing the manual vs non manual occupation variable with first the full 7 category occupation variable and second, (vi) with a 4 category variable age leaving education (8-15, 16-17, 18-21 and 22+ years). (vii) confounding by common co-morbidities; adjusting for type 2 diabetes, atrial fibrillation and chronic kidney disease (viii) additional adjustments for lipids and blood pressure which we would expect to be on the causal pathway between PA and CVD. The latter three analyses were conducted with and without use of multiple imputation for missing co-variate data, to assess the impact of missing data. 77% of participants had complete case data, the greatest amount of missing data was for atrial fibrillation (11%), education (8%) and CKD (7%), other covariates had <3 % missing data. Multiple imputation with chained equations was used and final analyses are combined estimates from 20 iterations^1^.

We investigated interactions firstly between sedentary behaviour and PA level, and secondly between each of MVPA, LIPA and sedentary behaviour and (i) age (above or below mean age of 78 years) and (ii) adiposity, above or below BMI of 28 Kg/m^2^ (iii) presence of disability.

**Sensitivity analyses: Results**

Analyses in Table 2 were repeated using square root transformed MVPA and the patterns of results observed were similar to untransformed data; coefficients for models 1-4 were significant. Analyses using percentage of the day spent in MVPA, LIPA and sedentary behaviour rather than minutes per day adjusted for wear time, did not change results (data not presented).

In analyses excluding 20 men who had a CVD event in the first year of follow-up, point estimates were similar to those in Table 2 and overall the pattern of results were the same, point estimates were similar although the confidence intervals for the continuous MVPA coefficient in model 4 and for the continuous SB coefficient in models 3 and 4 included 1. In analyses excluding men with mobility limitations, the first year of follow up and prevalent CVD (sample reduces to n=948), coefficients were similar but some confidence intervals were a little wider. In analyses where model 3 was repeated adjusted first for the 7 categories of social class and secondly for age at leaving education, coefficients remained very similar. Additional adjustments for LDL-cholesterol, use of statins, systolic blood pressure and use of anti-hypertensives little changed the coefficients for associations between PA and sedentary behaviour and onset of CVD, except that in model 4 the association between MVPA and CVD was attenuated by adjustment for sedentary time. Adjustment for presence of important co-morbidites (type 2 diabetes, chronic kidney disease and atrial fibrillation) also little changed coefficients. The analyses using multiple imputation for the co-variate missing data provided similar results, although again in model 4 the association between MVPA and CVD was attenuated by adjustment for sedentary time.

We tested whether the associations between sedentary behaviour and CVD differed in the above median active (32 minutes MVPA per day) vs below median active and observed no evidence for an interaction (LRT, p=0.66) Likewise, we did not find evidence that associations between MVPA, LIPA or sedentary behaviour and CVD varied by age, overweight status or disability.

**Supplementary Table 1**

Characteristics of men (self-reported 10 years prior to Actigraph survey) with and without Actigrpah data, n=3137 men.

|  | Without Actigraph | With Actigraph | Total | P (difference) |
| --- | --- | --- | --- | --- |
| Total N | 1571 | 1566 | 3137 |  |
| Non–manual social class at baseline, % (n) | 41.2 (627) | 52.65 (816) | 47.0 (1443) | <0.0001 |
| 20 year follow up characteristics |  |  |  |  |
| Age at survey (years), mean (SD) | 68.3 (5.2) | 66.2 (4.6) | 2597 | <0.0001 |
| Physical activity level, % (n) |  |  |  | <0.0001 |
| Inactive | 8.7(96) | 5.7(80) | 7.0(176) |  |
| Occasional activity | 25.6(283) | 18.0(254) | 21.3(537) |  |
| Light activity | 21.3(236) | 17.7(250) | 19.3(486) |  |
| Moderate activity | 14.2(157) | 17.9(252) | 16.2(409) |  |
| Vigorous activity | 15.5(171) | 20.8(294) | 18.5(465) |  |
| Very vigorous activity | 14.8(164) | 20.0(282) | 17.7(446) |  |
| Weight (Kg), mean (SD) | 81.0 (13.2) | 80.3(11.2) | 80.1(12.6) | 0.010 |
| Height (cm) , mean (SD) | 172.4 (6.6) | 173.1 (6.3) | 172.3 (6.5) | 0.210 |

**Supplementary Table 2**

**Distribution of bouts of moderate to vigorous PA, light PA and SB, and breaks in sedentary time among British Men Without Pre-Existing CVD or Heart Failure, (n=1274).**

|  | Mean Minutes/day | % of total average daily wear time in bouts |
| --- | --- | --- |
| Bouts of MVPA (>1040 CPM) |  |  |
| 1-9 minutes | 30.3 | 3.5 |
| >=10 minutes | 10.0 | 1.1 |
| Bouts of LIPA (100-1040 CPM) |  |  |
| 1-9 minutes | 176.5 | 20.6 |
| >=10 minutes | 22.0 | 2.6 |
| Bouts of Sedentary behaviour (<100 CPM) |  |  |
| 1-15 minutes | 222.4 | 25.9 |
| 16-30 minutes | 128.3 | 15.0 |
| 31-60 minutes | 148.1 | 17.4 |
| >=61 minutes | 117.2 | 13.9 |

MVPA, moderate and vigorous physical activity, LIPA, light activity

**Supplementary Table 3. Characteristics of British Men Without Pre-Existing CVD or Heart Failure, by Quartile of Daily Minutes Spent in MVPA, Measured in 2010-2012, (n=1274).**

|  | **Quartile of MVPA (minutes/day)** | | | |  |  |  |
| --- | --- | --- | --- | --- | --- | --- | --- |
|  | **1** | **2** | **3** | **4** |  | **All men** |  |
| Mean (SD) or % (n) | 0.4 – <3.1 | ≥3.1 - <30.8 | ≥30.8 – <53.5 | ≥53.5 | ***P* (trend)** |  | **N** |
| N | 291 ^a^ | 308 ^a^ | 340 ^a^ | 335 ^a^ |  |  | 1274 |
| Age (years) | 81.0 (5.0) | 78.7 (4.7) | 77.8 (4.0) | 76.5 (3.5) | <0.0001 | 78.4 (4.6) | 1274 |
| Manual Social class, % (n) | 52 (150) | 45 (139) | 45 (154) | 46 (151) | 0.29^b^ | 46.9 (594) | 1266 |
| Lives alone, % (n) | 23 (65) | 19 (59) | 19 (62) | 16 (52) | 0.18^b^ | 19.0 (238) | 1256 |
| Smoker, % (n) | 6.6 (19) | 4.6 (14) | 1.5 (5) | 2.1 (7) | 0.002^c^ | 3.6 (45) | 1257 |
| Alcohol (units per week) | 5.2 (7.3) | 6.0 (7.7) | 6.8 (7.5) | 7.2 (7.9) | <0.0001 | 6.4 (7.6) | 1240 |
| BMI (Kg/m^2^) | 28.2 (4.6) | 27.4 (3.6) | 26.9 (3.6) | 26.1 (3.1) | <0.0001 | 27.1 (3.8) | 1263 |
| LDL- cholesterol (mmol/L) | 2.4 (0.9) | 2.7 (0.9) | 2.7 (0.9) | 2.9 (1.0) | <0.0001 | 2.7 (0.9) | 1205 |
| Taking statins, %, (n) | 54.2 (174) | 43.8 (139) | 42.3 (135) | 38.8 (132) | 0.001 | 44.8 (571) | 1274 |
| Systolic Blood Pressure (mmHg) | 143.7 (20.5) | 147.1 (18.6) | 148.6 (17.4) | 150.3 (17.9) | <0.0001 | 147.4 (18.8) | 1271 |
| Taking antihypertensives, % (n) | 67.3 (216) | 53.0(168) | 52.6 (168) | 43.5 (138) | <0.0001 | 54.2 (690) | 1274 |
| Atrial Fibrilliation, % (n) | 12.5 (33) | 8.2 (23) | 7.2 (21) | 5.2 (15) | 0.015 | 8.2 (92) | 1127 |
| Chronic Kidney Disease: eGFR <45 ml/min per 1.73 m^2,^ % n | 18.1 (52) | 12.0 (35) | 3.3(10) | 3.3 (10) | <0.0001 | 9.1 (107) | 1183 |
| Type 2 Diabetes, % (n) | 21.6(63) | 10.7(33) | 14.4(49) | 10.1(34) | <0.0001 | 14.1(179) | 1273 |
| Mobility disability present, % (n) | 48.8 (139) | 14.3 (44) | 7.2 (24) | 6.4 (21) | <0.0001 | 18.2 (228) | 1253 |
| Sleep per night (hours) | 6.8 (1.5) | 6.9 (1.4) | 6.8 (1.3) | 6.9 (1.2) | 0.32 | 6.9 (1.4) | 1245 |
|  |  |  |  |  |  |  |  |
| Total activity (counts per minute) | 61,669  (24,590) | 113,645 (23,416) | 171,554 (29,976) | 294,370  (83,994) | <0.0001 | 164,749 (99271) | 1274 |
| Steps/day | 1895 (883) | 3646 (832) | 5302 (1022) | 8401 (2370) | <0.0001 | 4938 (2794) | 1274 |
| % time spent sedentary | 81.8 (6.7) | 75.1 (5.6) | 70.4 (5.7) | 63.0 (7.5) | <0.0001 | 72.2 (9.3) | 1274 |
| % time LIPA | 17.3 (6.5) | 22.2 (5.5) | 24.8 (5.7) | 27.3 (6.5) | <0.0001 | 23.1 (7.0) | 1274 |
| % time MVPA | 0.8 (0.4) | 2.6 (0.6) | 4.8 (0.8) | 9.7 (3.1) | <0.0001 | 4.7 (3.7) | 1274 |
| Sedentary behaviour (mins/day) | 676 (76) | 638 (65) | 607 (68) | 552 (76) | <0.0001 | 616 (84) | 1274 |
| LIPA (mins/day) | 144 (56) | 189 (50) | 214 (52) | 239 (61) | <0.0001 | 199 (65) | 1274 |
| MVPA (mins/day) | 6.9 (3.7) | 22.3 (4.8) | 41.4 (6.5) | 84.7 (26.9) | <0.0001 | 40 (33) | 1274 |
| Sedentary breaks (median, IQR)^e^ | 5.8 (4.6-6.8) | 6.8(5.9-7.9) | 7.4(6.5-8.7) | 8.4(6.9-9.6) | <0.0001 | 7.0 (5.9-8.5) | 1274 |

BMI: body mass index, LIPA: light physical activity, MVPA: moderate and vigorous physical activity, sedentary behaviour: sedentary behaviour eGFR: estimated Glomerular Filtration Rate.

^a^maximum N in quartile, varies slightly with missing covariate data

^b^Pearson chi square test

^c^Fisher’s exact test
^d^ geometric mean
^e^median and interquartile range of the number of breaks in sedentary time per hour

**Supplementary Table 4**

**Akaike Information Criteria (AIC) for Linear Compared to Non-Linear Models of Physical Activity in Relation to CVD events**

|  | Degrees of freedom | AIC | Degrees of freedom | AIC | Difference in AIC |
| --- | --- | --- | --- | --- | --- |
|  | Linear model | | Penalised spline model | |  |
| Steps/day | 1 | 1626 | 3 | 1605 | 19 |
| Sedentary minutes/day | 1 | 1633 | 3 | 1613 | 20 |
| Light PA minutes/day | 1 | 1638 | 3 | 1614 | 24 |
| MVPA minutes/day | 1 | 1628 | 3 | 1601 | 27 |

**Supplementary Table 5**

**Association Between achieving 150 minutes/week of Moderate to Vigorous Physical Activity (MVPA) in (a) Sporadic Minutes And (b) Bouts Lasting >=10 Minutes With CVD events, (n=1181 Men).**

|  | <150 minutes/week in bouts ≥1 minute | ≥150 minutes/week in bouts ≥1 minute | |  | <150 minutes/week in bouts ≥10 minutes | | | ≥150 minutes/week in bouts ≥10 minutes | |  |  |
| --- | --- | --- | --- | --- | --- | --- | --- | --- | --- | --- | --- |
| N Participants (n CVD events) | 431(71) | 843(66) | |  | 1074(127) | | | 200(10) | |  |  |
| Person years | 1811 | 3967 | |  | 4826 | | | 952 | |  |  |
| CVD events/ 1000 person years | 31.1 | 16.6 | |  | 26.3 | | | 10.5 | |  |  |
|  | | HR | 95%CI |  |  | | | HR | 95%CI | |  |
| Model 1^a^ | Reference | **0.47** | **0.32, 0.69** |  | | Reference | **0.49** | | **0.25, 0.98** | | |
| Model 2^b^ | Reference | **0.51** | **0.34, 0.76** |  | | Reference | **0.55** | | **0.27, 1.09** | | |
| Model 3^c^ | Reference | **0.55** | **0.36,0.84** |  | | Reference | **0.57** | | **0.28, 1.14** | | |
|  |  |  |  |  | |  |  | |  | | |

^a^model 1=age+ region of residence+ season of wear+ accelerometer wear time

^b^model 2=model 1+ social class+ alcohol use+ smoking+ sleep time+ living alone+ BMI

^c^model 3=model 2+ mobility disability

**Supplementary Table 6 Association Between Duration of Bouts of Sedentary Behaviour, Light PA and MVPA^a^ With CVD events, (n=1181 Men).**

|  | Bouts of 1-9 minutes | | Bouts of >=10 minutes | |  |  |  |  | P^e^ |
| --- | --- | --- | --- | --- | --- | --- | --- | --- | --- |
| MVPA | HR^f^ | 95% CI | HR^f^ | 95% CI | HR^f^ | 95%CI | HR^f^ | 95%CI | 0.750 |
| Model 1^b^ | 0.99 | (0.97, 1.00) | 0.99 | (0.97, 1.01) |  |  |  |  |  |
| Model 2^c^ | 0.99 | (0.97, 1.00) | 0.99 | (0.97, 1.01) |  |  |  |  |  |
| Model 3^d^ | 0.99 | (0.97, 1.00) | 0.99 | (0.97, 1.01) |  |  |  |  |  |
| LIPA |  |  |  |  |  |  |  |  |  |
| Model 1^b^ | 0.99 | (0.99,1.00) | 1.00 | (0.99, 1.01) |  |  |  |  | 0.440 |
| Model 2^c^ | 0.99 | (0.99,1.00) | 1.00 | (0.99, 1.01) |  |  |  |  |  |
| Model 3^d^ | 0.99 | (0.99,1.00) | 1.00 | (0.99, 1.01) |  |  |  |  |  |
| Sedentary Behaviour | 1-15 minutes | | 16-30, minutes | | 31-60 minutes | | >61 minutes | |  |
| Model 1^b^ | 1.00 | (1.00,1.01) | 1.00 | (1.00,1.01) | 1.01 | (1.00,1.01) | 1.00 | (1.00,1.01) | 0.972 |
| Model 2^c^ | 1.00 | (1.00,1.01) | 1.00 | (1.00,1.01) | 1.01 | (1.00,1.01) | 1.00 | (1.00,1.01) |  |
| Model 3^d^ | 1.00 | (1.00,1.01) | 1.00 | (1.00,1.01) | 1.01 | (1.00,1.01) | 1.00 | (1.00,1.01) |  |

^a^The number of minutes/day in bouts of the specified duration. HR is per minute of activity in the specified bout duration.
^b^Model 1=age+ region of residence+ season of wear+ accelerometer wear time+ minutes of sedentary behaviour
^c^Model 2=model 1+ social class+ alcohol use+ smoking+ sleep time+ living alone+ BMI
^d^Model 3=model 2+ mobility disability
^e^test for no difference between bouts durations
^f^HR per minute in bout of specified duration. Bold font; p<0.05

**Supplementary Table 7 Association Between number of Sedentary Breaks per Hour^a^ With CVD events, (n=1181 Men).**

|  | **Quartile 1**  (0.3-5.8) | **Quartile 2**  (5.9-6.9) | | | **Quartile 3**  (7.0- 8.4) | | **Quartile 4**  (8.5- 15.9) | | | **Total** | |
| --- | --- | --- | --- | --- | --- | --- | --- | --- | --- | --- | --- |
| N Participants (n deaths) | 285 (41) | 301 (32) | | | 298 (25) | | 297 (24) | | | 1181 (122) | |
| Person years | 1710 | 1365 | | | 1412 | | 1431 | | | 5378 | |
| CVD events/ 1000 person years | 35.0 | 23.5 | | | 17.7 | | 16.8 | | | 22.6 | |
|  | | HR^e^ | 95%CI | | HR^e^ | 95%CI | HR^e^ | 95%CI | |  |  |
| Model 1^b^ | Reference | 0.91 | | (0.54,1.52) | 0.94 | (0.50,1.79) | 1.35 | | (0.60,3.03) |  |  |
| Model 2^c^ | Reference | 0.94 | | (0.56,1.59) | 1.02 | (0.54,1.96) | 1.44 | | (0.64,3.24) |  |  |
| Model 3^d^ | Reference | 0.94 | | (0.56,1.58) | 1.01 | (0.52,1.93) | 1.36 | | (0.60,3.09) |  |  |

^a^A sedentary break is the interruption of a sedentary bout lasting >1 minute by ≥1 minute of LIPA or MVPA
^b^Model 1=age +region of residence+ season of wear+ accelerometer wear time+ minutes of sedentary behaviour
^c^Model 2=model 1+ social class+ alcohol use+ smoking+ sleep time+ living alone+ BMI
^d^Model 3=model 2+ mobility disability
^e^HR is per quartile of sedentary breaks per hour.

**Web Figure 1**

Web Figure 2

Web Figure 3

**
Web Figure 4**

1. White IR, Royston P, Wood AM. Multiple imputation using chained equations: Issues and guidance for practice. *Statistics in Medicine* 2010;30:377-99. doi: 10.1002/sim.4067
